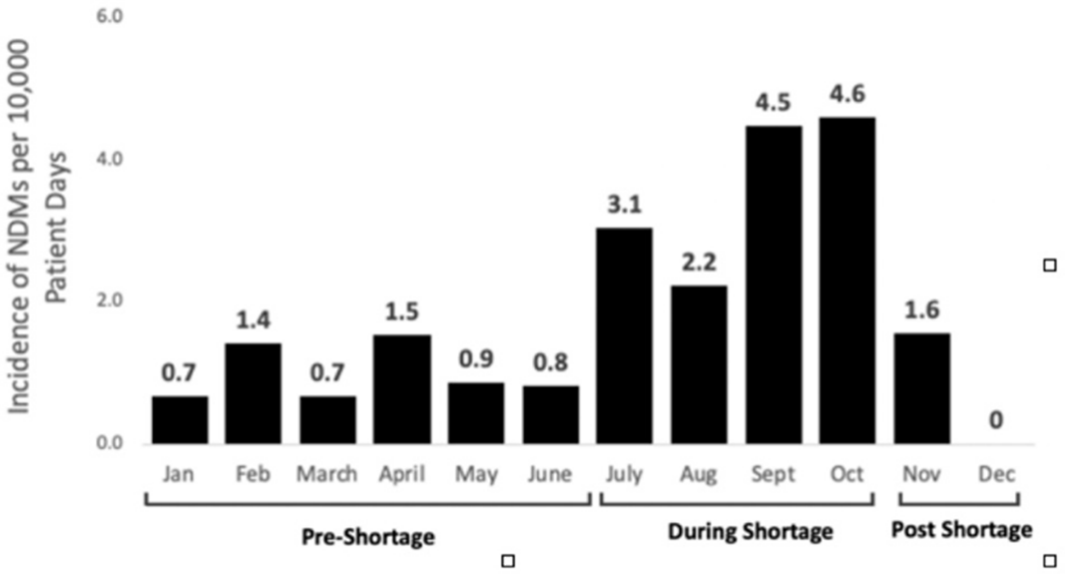# Retrospective Study on Personal Protective Equipment During Pandemic Link to Outbreak of Carbpenem-Resistant Enterobacteriace

**DOI:** 10.1017/ash.2021.17

**Published:** 2021-07-29

**Authors:** Kenisha Evans, Jennifer LeRose, Angela Beatriz Cruz, Lavina Jabbo, Teena Chopra

## Abstract

**Background:** In 2019, according to the Centers for Disease Control and Prevention, carbapenem-resistant Enterobacteriaceae (CRE), had cost the lives of >35,000 patients, particularly the most virulent plasmid-mediated New Delhi metallo-β-lactamase (NDM). Although healthcare systems normally have strict surveillance and infection control measures for CRE, the rapid emergence of novel SAR-CoV-2 and COVID-19 led to a shortage of personal protective equipment (PPE) and medical supplies. As a result, routine infection practices, such as contact precautions, were violated. Studies have shown this depletion and shift in resources compromised the control of infections such CRE leading to rising horizontal transmission. **Method:** A retrospective study was conducted at a tertiary healthcare system in Detroit, Michigan, to determine the impact of PPE shortages during the COVID-19 pandemic on NDM infection rates. The following periods were established during 2020 based on PPE availability: (1) pre-PPE shortage (January–June), (2) PPE shortage (July–October), and (3) post-PPE shortage (November–December). Rates of NDM per 10,000 patient days were compared between periods using the Wilcoxon signed rank-sum test. Isolates were confirmed resistant by NDM by molecular typing performed by the Michigan State Health Department. Patient characteristics were gathered by medical chart review and patient interviews by telephone. **Results:** Overall, the average rate of NDM infections was 1.82 ±1.5 per 10,000 patient days. Rates during the PPE shortage were significantly higher, averaging 3.6 ±1.1 cases per 10,000 patient days (*P* = .02). During this time, several infections occurred within patients on the same unit and/or patients with same treating team, suggesting possible horizontal transmission. Once PPE stock was replenished and isolation practices were reinstated, NDM infection rates decreased to 0.77 ±1.1 per 10,000 patient days. **Conclusion:** Control of CRE requires strategic planning with active surveillance, antimicrobial constructs, and infection control measures. The study illustrates that in times of crisis, such as the COVID-19 pandemic, the burden of effective infection control requires much more multidisciplinary efforts to prevent unintentional lapses in patient safety. A swift response by the state and local health departments at a tertiary-care healthcare center conveyed a positive mitigation of the highest clinical threats and decreased horizontal transmission of disease.

**Funding:** No

**Disclosures:** None

**Figure 1.**